# Molecular Mechanisms Involved in Sperm Development, Maturation, and Fertilization

**DOI:** 10.3390/ijms26094049

**Published:** 2025-04-25

**Authors:** Manuel Álvarez-Rodríguez, Jaime Catalán

**Affiliations:** 1 Department of Animal Reproduction, Spanish National Institute for Agricultural and Food Research and Technology (INIA-CSIC), 28040 Madrid, Spain; 2 Biotechnology of Animal and Human Reproduction (TechnoSperm), Institute of Food and Agricultural Technology, University of Girona, 17003 Girona, Spain; 3 Unit of Cell Biology, Department of Biology, Faculty of Sciences, University of Girona, 17003 Girona, Spain

The journey of the spermatozoon, from its formation in the testis to its fusion with the oocyte, is a process carefully regulated by an intricate network of molecular mechanisms. These mechanisms are essential to ensure the functional competence of the male gamete and its ability to generate viable offspring. As studies on sperm physiology advance, it has become evident that sperm must not only be able to swim and reach the oocyte but also interact in a highly sophisticated manner with their environment, both in the male and female reproductive tracts. In this regard, the development of high-performance technologies and equipment, integrative molecular approaches, and in vitro models has greatly enhanced knowledge about the mechanisms underlying male fertility.

This Special Issue, in both of its versions, presents a valuable collection of original articles and reviews that address key aspects of sperm biology, highlighting the importance of molecular factors, cellular interactions, and reproductive environmental conditions in sperm quality and fertilization. It also provides novel evidence on how genetic, epigenetic, biochemical, immunological, and environmental factors influence sperm physiology.

Among the original articles, several studies emphasize the molecular and cellular regulation of sperm function and male fertility. While one of the investigations on sperm chromatin condensation revealed that defects in this process accelerate early embryonic development without altering ICSI outcomes [1], another study on sperm chromatin status, conducted in mouse species with divergent mating systems, revealed differences in DNA fragmentation among them. The authors suggested that the relationship between chromatin status and DNA integrity may be linked to levels of sperm competition and offered an evolutionary perspective on sperm quality and selective pressures [2]. Additionally, a study investigating a gene encoding a histone from the H2B family identified a functional association between H2BFWT gene variants in human sperm and pregnancy outcomes following ICSI treatment [3]. Altogether, these findings reinforce the importance of genomic research in investigating the genetic causes of male infertility.

Advancements in reproductive technologies are also highlighted in this Special Issue by showcasing the methods available for the optimization of semen cryopreservation and sperm selection protocols. A comparison between slow freezing and vitrification of human semen showed significant differences in post-thaw quality and the expression of miRNAs related to cellular stress [4]. In another study, a protocol for equine sperm sex classification based on absolute RT-qPCR was successfully standardized. This represents progress in non-invasive and more precise sperm selection in animals and demonstrates the potential of molecular approaches to improve assisted reproduction in non-human species [5].

Environmental and physiological modulators of fertility are also addressed. A study in rats showed that moderate and sustained lifelong physical exercise does not prevent age-related testicular degeneration and may even activate cellular stress pathways and inhibit protein synthesis, thereby compromising mitochondrial function [6]. Furthermore, it was found that exposure to non-physiological temperatures negatively affects spermatogenesis progression and compromises Sertoli cell function, highlighting the testis’s sensitivity to thermal conditions [7]. The evaluation of oxidation–reduction potential (ORP) in semen as an indicator of oxidative stress revealed an association between elevated ORP levels and decreased sperm quality, although the authors emphasized the need for further standardization before clinical application [8].

From a developmental perspective, some studies in this collection of recent research on male fertility shed light on the complex regulation of spermatogenesis and early embryogenesis. For example, dynamic changes in the formation of R-loops during zygotic genome activation in mouse embryos were identified, pointing to these DNA-RNA hybrids as essential regulators in the early stages of development [9]. Another study showed that IL-1β acts as a regulator of spermatogenesis in vitro in murine models treated with busulfan, demonstrating the effect of its addition on the promotion of germ cell differentiation, opening therapeutic possibilities for individuals with physiological or pathological testicular conditions that can impair male fertility [10]. Other articles highlight the role of specific molecular players during fertilization, such as the sperm membrane protein SMA20/PMIS2, which may have divergent functions between species [11], or β integrin, which has been found to play a more significant role in later stages of reproduction, as its absence in gametes, despite not preventing fertilization, compromises post-implantation embryonic development [12].

Two studies focused specifically on the interaction between the sperm and the oviductal environment. One revealed that the mature cumulus–oocyte complex promotes NPPC production in the ampulla, which is necessary to release spermatozoa from the epithelial cells of the porcine oviduct [13]. The other indicated that late calcium oscillation in mouse oocytes causes polyspermy, although it may still allow for normal embryonic development [14]. These findings reinforce the active role of the female microenvironment in sperm selection and functionality.

Among the innovative approaches, one study using a virtual screening and molecular docking approach identified the pesticide famoxadone as a potential reproductive disruptor due to its affinity for a set of proteins involved in gamete interaction and zygote development. This highlights the importance of evaluating the environmental impact of emerging contaminants on fertility [15]. Another emerging area of interest is the role of the seminal microbiome in idiopathic male infertility, as explored in this Special Issue through third-generation sequencing platforms [16].

The review articles in this collection provide integrative frameworks to understand sperm physiology. A systematic review and meta-analysis on *H19* gene methylation patterns found consistently lower levels in infertile men compared to fertile controls, supporting its potential as an epigenetic marker in diagnosing male infertility [17]. The complex regulation of sperm motility through the interaction of phosphatases PP1, PP2A, and PP2B is also described [18]. Another review showcased the toxicology of polybrominated diphenyl ethers (PBDEs), describing their disruptive effects on various reproductive parameters such as hormone production and sperm quality in both developing and adult individuals and emphasizing their persistence in the environment as a significant reproductive health risk [19]. At the molecular level, another study highlighted the role of Tubulin Polymerization-Promoting Protein 2 (TPPP2) in spermatogenesis, focusing on its regulatory implications [20]. Finally, a transcriptomics-based review mapped the progress of human spermatogenesis, providing a comprehensive reference of testicular development and maturation biology from the fetal period to adulthood for potential use in clinical and diagnostic applications [21].

The opinion article included in this Special Issue highlighted the central role of cyclic adenosine monophosphate (cAMP) in gamete development and fertilization. Beyond its classical signaling function, cAMP emerges as a potential target for infertility treatment [22].

In closing, this Special Issue reflects the multidisciplinary nature of current research on sperm physiology, combining molecular biology, genomics, environmental sciences, and reproductive technologies. We are confident that the findings presented here will inspire future research and contribute to the advancement of reproductive medicine.
